# Cobalt Bis-Dicarbollide Enhances Antibiotics Action towards *Staphylococcus epidermidis* Planktonic Growth Due to Cell Envelopes Disruption

**DOI:** 10.3390/ph15050534

**Published:** 2022-04-26

**Authors:** Eva Vaňková, Kristýna Lokočová, Petra Kašparová, Romana Hadravová, Ivana Křížová, Olga Maťátková, Jan Masák, Václav Šícha

**Affiliations:** 1 Department of Biotechnology, University of Chemistry and Technology Prague, Technická 5, 166 28 Prague, Czech Republic; kristynalokocova@gmail.com (K.L.); petra.kasparova@vscht.cz (P.K.); ivana.krizova@vscht.cz (I.K.); olga.matatkova@vscht.cz (O.M.); jan.masak@vscht.cz (J.M.); 2 Institute of Organic Chemistry and Biochemistry of the Czech Academy of Sciences, Flemingovo náměstí 542/2, 166 10 Prague, Czech Republic; romana.hadravova@uochb.cas.cz; 3 Chemistry Department, Faculty of Science, Jan Evangelista Purkyně University in Ústí nad Labem, Pasteurova 15, 400 96 Ústí nad Labem, Czech Republic; vaclav.sicha@ujep.cz

**Keywords:** additive effect, antibiotics, antimicrobial activity, carborane, erythromycin, Gram-positive bacterium, metallacarboranes, synergistic effect, tetracycline, vancomycin

## Abstract

The emergence of antibiotic resistance in opportunistic pathogens represents a huge problem, the solution for which may be a treatment with a combination of multiple antimicrobial agents. Sodium salt of cobalt bis-dicarbollide (COSAN.Na) is one of the very stable, low-toxic, amphiphilic boron-rich sandwich complex heteroboranes. This compound has a wide range of potential applications in the biological sciences due to its antitumor, anti-HIV-1, antimicrobial and antibiofilm activity. Our study confirmed the ability of COSAN.Na (in the concentration range 0.2–2.48 µg/mL) to enhance tetracycline, erythromycin, and vancomycin action towards *Staphylococcus epidermidis* planktonic growth with an additive or synergistic effect (e.g., the combination of 1.24 µg/mL COSAN.Na and 6.5 µg/mL TET). The effective inhibitory concentration of antibiotics was reduced up to tenfold most efficiently in the case of tetracycline (from 65 to 6.5 µg/mL). In addition, strong effect of COSAN.Na on disruption of the cell envelopes was determined using propidium iodide uptake measurement and further confirmed by transmission electron microscopy. The combination of amphiphilic COSAN.Na with antibiotics can therefore be considered a promising way to overcome antibiotic resistance in Gram-positive cocci.

## 1. Introduction

The opportunistic pathogen *Staphylococcus epidermidis* colonizes human skin and stands for one of the most common causative agents of nosocomial infections. The ability of these pathogens to cause infection is strictly linked to compromised immunity, and thus they affect, e.g., HIV-positive or oncological patients [1]. The invasive form is often characterized by increased activity of resistance mechanisms, thus the treatment of developed infections (with the possibility of forming a biofilm) in humans with conventional antibiotics can be extremely difficult [2,3]. Researchers are therefore trying to find new therapeutic strategies, especially with unusual substances that can help in the treatment of microbial infections and related diseases, while not creating selective pressure on the treated population and supporting the development of resistance in such process [4,5]. Previously published examples of known agents capable of enhancing the inhibitory effect of antibiotics are most often compounds isolated from natural sources, which include antimicrobial peptides [6,7], stilbenes [8,9,10], polysaccharides [11,12], alkaloids [13], aldehydes [14] or catechins [15]. 

Heteroboranes are inorganic complexes consisting primarily of boron hydrides with another element(s) such as carbon or metal [16]. These substances dispose of many important properties such as chemical and thermal stability [17], rigid geometry, delocalized negative charge, ion-pairing behavior [18], strong acidity of their conjugated acids [19], solubility in water and oils [20], ability to self-assembly into monolayer spherical nano-vesicles [21,22], non-specific interaction with the protein surface and high affinity to albumin [23,24]. The most studied area of application for heteroboranes in biological sciences is boron neutron capture therapy used for the treatment of locally invasive malignant tumors [23,25,26]. In addition, these substances have proven capability to inhibit some liver microsomal enzymes [27]. 

Functionalized inorganic three-dimensional clusters, including pharmaceutically important representatives of an orange cobalt(III) bis-(η^5^-dicarbollide) 6 π e- sandwich complex, (COSAN^-^)—3,3′-*commo*-bis[*closo*-1,2-dicarba-3-cobaltadodecaborate(11)](1-), are specific potent inhibitors of retroviral HIV-1 protease [28]. COSAN^-^ alkylsulfonamide derivatives also strongly and selectively inhibit carbonic anhydrase IX isoform, which is an enzyme expressed on the surface of cells in hypoxic tumors [29]. Sodium salt of cobalt bis-dicarbollide (COSAN.Na) itself and their alkyl- or arylammonium derivatives have selective antimicrobial [30,31,32,33] and antibiofilm properties [34]. Furthermore, these compounds are non-toxic to mammalian cells and, as amphiphilic molecules, are able to pass through the biological membranes [20,21]. 

The bactericidal and fungicidal activity of N-substituted *closo*- and *nido*-dicarbaboranes derivatives and related compounds was first described in 1970 [35]. Further studies brought positive information about the antimicrobial activity of various bis-*o*-dicarbaboranes, *closo*-*o*-dicarbaboranes, and *nido*-type dicarbollide anions towards the representatives of microscopic fungi and bacteria *Staphylococcus aureus*, *Streptococcus pyogenes*, *Escherichia coli*, *Klebsiella pneumoniae*, *Mycobacterium* spp., *Enterobacter cloacae*, *Pseudomonas aeruginosa*, *Acinetobacter baumannii*, etc. [36,37,38,39,40,41]. More information about these properties was published in an article focused on the antimicrobial activity of COSAN derivatives [32]. It was proven that these substances inhibit the microbial growth of *Candida* spp. and 16 other bacterial strains including *S. aureus*, *P. aeruginosa* and *E. coli*. Strong and selective antimicrobial activity of inorganic/organic fused boraheterocycles against *Neisseria gonorrhoeae* was also described [33]. Benkocká et al. [31] verified antimicrobial activity of COSAN^-^ derivatives (including algae) grafted on polystyrene foil. In 2017, our research group published an article pointing out the selective antimicrobial activity of COSAN^-^ derivatives on Gram-positive bacteria, especially *Staphylococcus* spp., and filamentous fungi *Trichosporon cutaneum* [30]. Later on, in 2019, we also proved the antibiofilm activity of COSAN^-^ and its two aminoderivatives towards Gram-positive bacteria (*S. aureus*, *S. epidermidis* and *Enterococcus faecalis*) as well as filamentous fungi *T. cutaneum*, while Gram-negative bacteria and *Candida* spp. biofilms were insensitive [34]. Only one other *o*-carborane-containing compound was studied as carborane antibiofilm agent until yet [42]. Carborane-based ferrocene-Ru(II)-arene complexes were used in synergy with nano-scaled TiO_2_ to overcome the multidrug resistance of bacteria *A. baumannii* [43]. Some substituted 8-iodine-cobalt bis-dicarbollide derivatives were synthetized and evaluated as effective against methicillin-resistant *S. aureus* (MRSA) [44], but also against Gram-negative bacteria, i.e., *P. aeruginosa* and *Yersinia enterocolitica* [45].

The present study was focused on the possibility of the enhancement of conventional antibiotics tetracycline (TET), erythromycin (ERM), and vancomycin (VAN) action against *S. epidermidis* planktonic growth by the addition of COSAN.Na. The main aim was to decrease the effective dose of antibiotics which is needed for efficient inhibition of *S. epidermidis* planktonic growth and thus overcome tolerance of the cells to antibiotics alone. The use of the combination of antibiotics and some unusual substances enable to maintain the use of antibiotics in medicine, and at the same time strengthen their inhibitory properties, which are often insufficient against constantly evolving microorganisms.

## 2. Results

### 2.1. Effect of Antibiotics or COSAN.Na on Planktonic Growth of S. epidermidis

The efficacy of COSAN.Na compared to the antibiotics TET, ERM and VAN in the inhibition of *S. epidermidis* planktonic growth was determined as minimum inhibitory concentrations that inhibit 80% of microbial growth (MIC_80_) compared to untreated samples (Table 1). The most effective antibiotic was ERM with MIC_80_ 0.5–0.75 µg/mL for all strains tested, MIC_80_ of VAN ranged from 1.5 to 2.75 µg/mL and in the case of TET, variable effectiveness was observed with MIC_80_ 5–65 µg/mL. The MIC_80_ of COSAN.Na was found in the concentration range 2–3.1 µg/mL, similarly to ERM and VAN action and much lower as compared to TET. According to the ratio of MIC_80_ and minimum bactericidal concentrations that inhibit any visible growth (MBC_99.9_) of COSAN.Na (MBC_99.9_/MIC_80_ < 4.0—bactericidal effect, MBC_99.9_/MIC_80_ > 4.0—bacteriostatic effect) shown in Table 1, COSAN.Na cannot be strictly classified as a bactericidal agent, because its effect was bactericidal in only one strain tested (*S. epidermidis* DBM 3179), while bacteriostatic in two others.

### 2.2. Combined Effect of Antibiotics with COSAN.Na on Planktonic Growth of S. epidermidis

The effect of the combination of COSAN.Na and each antibiotic (TET, ERM or VAN) on planktonic growth of different strains of *S. epidermidis* determined as relative percentages of OD_600nm_ I is depicted in detail in Figures S1–S3. 

As we can see in Figure S1, the most effective enhancement of antibiotic inhibitory action by COSAN.Na addition was achieved in the case of TET towards *S. epidermidis* DBM 3179 planktonic growth. Low subinhibitory concentrations of both substances in combination were able to inhibit the growth by at least 80% (Figure S1a). The most effective combination of TET (13 µg/mL) and COSAN.Na (0.62 µg/mL) for this strain had a synergistic effect according to the lowest fractional inhibitory concentration index (FICi; 0.4; Table 2). Concerning the requirement for the greatest reduction of an effective antibiotic concentration, the addition of 1.24 µg/mL COSAN.Na to TET lowered the needed concentration of TET to 6.5 µg/mL (Table 2). Therefore, we achieved a 10-fold reduction in a TET concentration (original MIC_80_ 65 µg/mL) required for 80% inhibition of *S. epidermidis* DBM 3179 planktonic growth. For the ERM and COSAN.Na, the most effective combination found (0.15 µg/mL ERM and 1.86 µg/mL COSAN.Na with FICi 0.8) had the additive effect only (Figure S1b, Table 2). Despite this, we also achieved the 10-fold decrease of needed concentration of ERM (original MIC_80_ 0.75 µg/mL) for 80% inhibition of *S. epidermidis* DBM 3179 planktonic growth by the addition of 2.48 µg/mL COSAN.Na to the 0.075 µg/mL ERM. However, it is evident that the addition of very low subinhibitory concentrations of COSAN.Na (0.31 and 0.62 µg/mL) to ERM had no significant effect on ERM inhibitory activity against *S. epidermidis* DBM 3179 (Figure S1b). The combination of VAN and COSAN.Na (Figure S1c) had a very similar character to a described combination of ERM and COSAN.Na. Their most effective combination according to FICi (0.15 µg/mL VAN and 1.86 µg/mL COSAN.Na with FICi 0.7) acted additively (Table 2). Nevertheless, their combination corresponding to the highest FICi corresponds also to the combination with the highest reduction in effective antibiotic concentration. In this case, we achieved again 10-fold decrease of the needed concentration of VAN (MIC_80_ 1.5 µg/mL) for 80% inhibition of *S. epidermidis* DBM 3179 planktonic growth.

The susceptibility of other tested strains of *S. epidermidis* to antibiotics and COSAN.Na combination was evaluated by the same way as previously described and the results are depicted in Figures S2 and S3. *S. epidermidis* CNCTC 5671 was the least sensitive to the action of combination of antibiotics TET, ERM or VAN and COSAN.Na (Figure S2, Table 2). The combination of antimicrobial agents used was additive in all cases. The planktonic growth of *S. epidermidis* CNCTC 5671 was inhibited by combination of TET and COSAN.Na as well as ERM and COSAN.Na with a similar trend (Figure S2). A concentration of 0.28 µg/mL COSAN.Na and 27 µg/mL TET with lowest FICi 0.7 caused 1.5-fold reduction of effective inhibitory concentration of TET. On the contrary, by adding 2.24 µg/mL COSAN.Na to 4.5 µg/mL TET, a 10-fold reduction of needed concentration of TET (original MIC_80_ 45 µg/mL) was achieved for 80% inhibition of *S. epidermidis* CNCTC 5671 planktonic growth. In the case of ERM and COSAN.Na, no combination of these agents effectively reduced the antibiotic concentration (a maximum reduction of 2.5-fold was achieved). However, compared to previously described *S. epidermidis* DBM 3179, the addition of subinhibitory concentrations of COSAN.Na to ERM significantly changed the inhibitory action of ERM against *S. epidermidis* CNCTC 5671 planktonic growth (Figure S2b). For VAN and COSAN.Na, only one very effective combination was found (0.15 µg/mL VAN and 2.24 µg/mL COSAN.Na) which had the lowest FICi 0.9 (acted additively) and simultaneously reduced 10-fold a concentration of VAN needed for 80% inhibition of *S. epidermidis* CNCTC 5671 planktonic growth.

In the case of *S. epidermidis* CCM 2343 (Figure S3), the combination of antimicrobial agents used was additive in all cases with the only exception of ERM (0.2 µg/mL) combined with COSAN.Na (0.2 µg/mL), which showed synergistic effect (FICi 0.5; Table 2). Nevertheless, the addition of COSAN.Na to TET or ERM helped reduce the effective concentration of antibiotics five times, which is still an important result. The combination of VAN and COSAN.Na did not have any apparent effect on inhibition of *S. epidermidis* CCM 2343 planktonic growth.

### 2.3. Cytotoxicity of Antibiotics and COSAN.Na Alone or in Their Combination

The cytotoxicity of antimicrobial agents used alone or in selected combinations towards human embryonic kidney cells (HEK 293T) cells is shown in Table S1. The antibiotics TET, ERM and VAN as well as COSAN.Na alone were tested at subinhibitory concentrations (which were used for their combination) or at MIC_80_. No cytotoxic concentration of these substances was found. HEK 293T cells survived in the presence of tested substances from more than 96% in all cases, and COSAN.Na did not show higher cytotoxicity than the investigated antibiotics. The most effective combinations of antibiotics and COSAN.Na against *S. epidermidis* CNCTC 5671 planktonic growth (shown in Table 2) were selected for cytotoxicity testing. Even in this case, no cytotoxic combination was found, and these combinations of antimicrobials are therefore considered to be gentle to HEK 293T cells.

### 2.4. Permeabilization Activity of Antibiotics and COSAN.Na Alone or in Their Combination against S. epidermidis CNCTC 5671 Cells

**Figure 1:**
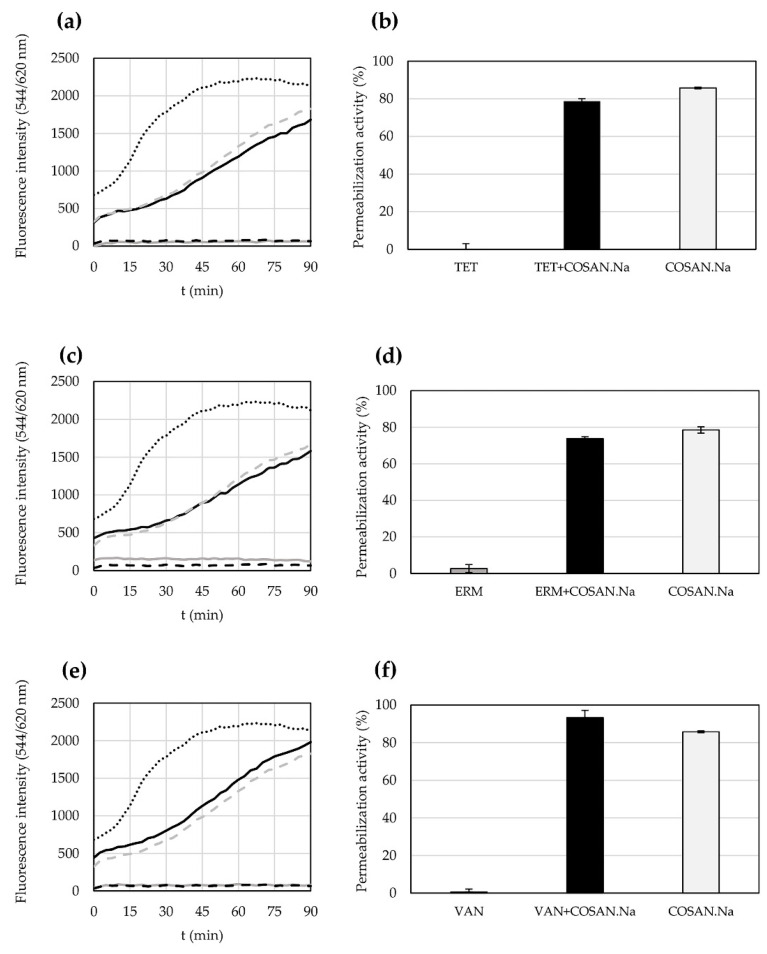
Cytoplasmic membrane permeabilization of *Staphylococcus epidermidis* CNCTC 5671 cells by tetracycline (TET), erythromycin (ERM), vancomycin (VAN), sodium salt of cobalt bis-dicarbollide (COSAN.Na) or their combination determined as fluorescence intensity of uptaken propidium iodide (PI). (**a**) PI uptake by cells affected by TET 4.5 µg/mL, COSAN.Na 2.24 µg/mL or TET 4.5 µg/mL + COSAN.Na 2.24 µg/mL; (**b**) permeabilization activity of TET 4.5 µg/mL, COSAN.Na 2.24 µg/mL or TET 4.5 µg/mL + COSAN.Na 2.24 µg/mL; (**c**) PI uptake by cells affected by ERM 0.3 µg/mL, COSAN.Na 1.68 µg/mL or ERM 0.3 µg/mL + COSAN.Na 1.68 µg/mL; (**d**) permeabilization activity of ERM 0.3 µg/mL, COSAN.Na 1.68 µg/mL or ERM 0.3 µg/mL + COSAN.Na 1.68 µg/mL; (**e**) PI uptake by cells affected by VAN 0.15 µg/mL, COSAN.Na 2.24 µg/mL or VAN 0.15 µg/mL + COSAN.Na 2.24 µg/mL; (**f**) permeabilization activity of VAN 0.15 µg/mL, COSAN.Na 2.24 µg/mL or VAN 0.15 µg/mL + COSAN.Na 2.24 µg/mL; black dotted lines indicate positive control (cell suspension mixed with octenidine dihydrochloride); black dashed lines indicate negative control (cell suspension in phosphate-buffered saline); grey lines indicate appropriate antibiotic at described concentrations; grey dashed lines indicate COSAN.Na at described concentrations; black lines indicate combination of appropriate antibiotic and COSAN.Na at described concentrations.

The ability of antibiotics (TET, ERM and VAN), COSAN.Na and their combination to permeabilize cytoplasmatic membrane of *S. epidermidis* CNCTC 5671 cells is depicted in Figure 1. As expected, the measured fluorescence intensity of uptake propidium iodide (PI) (544/620 nm) as well as the calculated permeabilization activity given in percentages confirmed that there was no disruption of *S. epidermidis* CNCTC 5671 cell membrane in the presence of used antibiotics. On the contrary, COSAN.Na had significant permeabilization activity even at subinhibitory concentrations (1.68 and 2.24 µg/mL) when used alone. A mixture of TET or ERM and COSAN.Na (at concentrations effective against *S. epidermidis* CNCTC 5671 planktonic growth according to Table 2) retained the permeabilization activity of COSAN.Na reaching almost 80%. The only exception showing an increase in permeabilization activity (to 93%) of antimicrobial agents’ mixture used was VAN and COSAN.Na (Figure 1e,f).

### 2.5. Morphology of S. epidermidis CNCTC 5671 Cells Affected by TET, COSAN.Na or Their Combination

**Figure 2:**
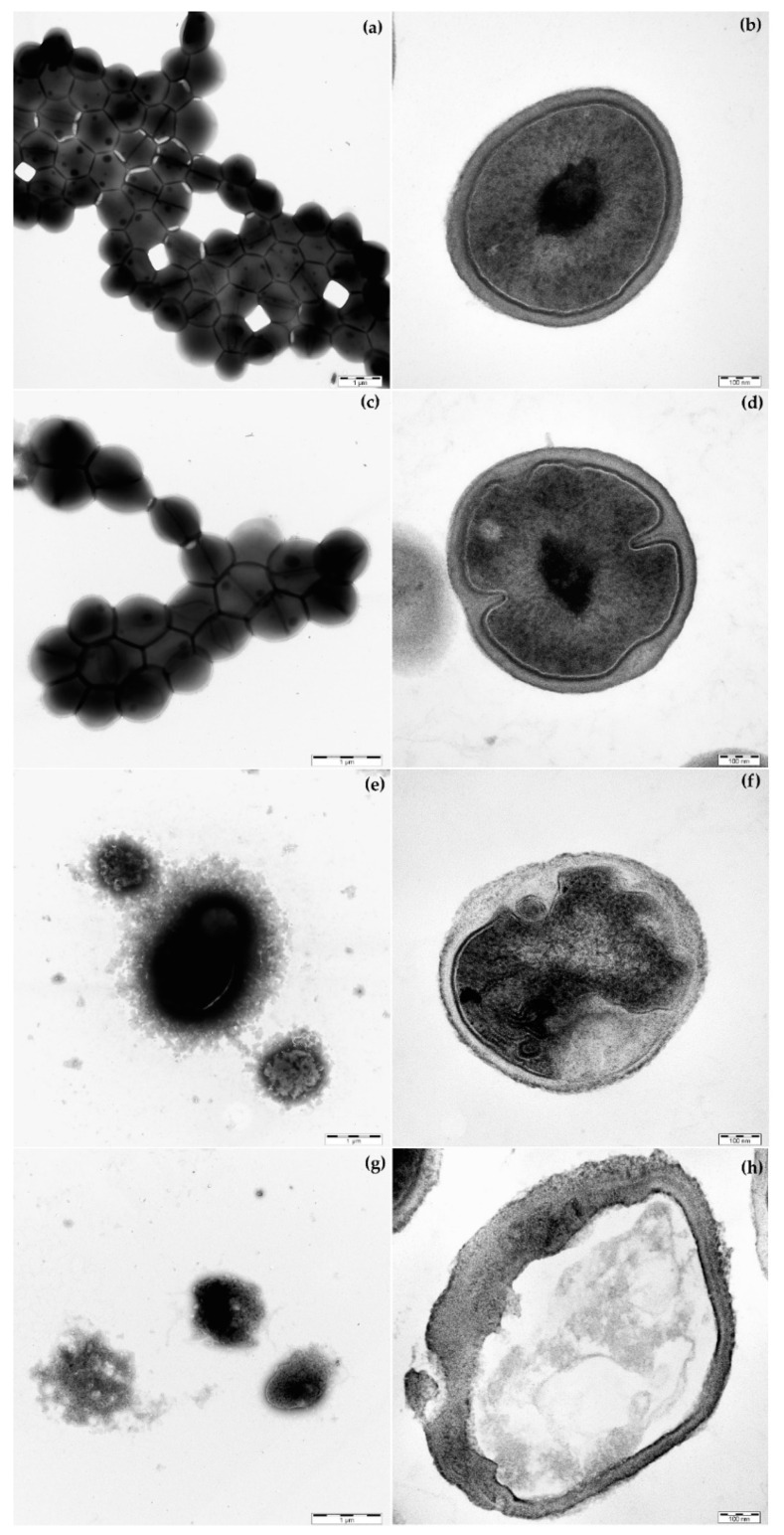
Transmission electron microscopy images of *Staphylococcus epidermidis* CNCTC 5671 affected by tetracycline (TET) and sodium salt of cobalt bis-dicarbollide (COSAN.Na) alone or their combination. (**a**) untreated control sample—negative staining; (**b**) untreated control sample—ultra-thin section; (**c**) TET 4.5 µg/mL—negative staining; (**d**) TET 4.5 µg/mL—ultra-thin section; (**e**) COSAN.Na 2.24 µg/mL—negative staining; (**f**) COSAN.Na 2.24 µg/mL—ultra-thin section; (**g**) TET 4.5 µg/mL + COSAN.Na 2.24 µg/mL—negative staining; (**h**) TET 4.5 µg/mL + COSAN.Na 2.24 µg/mL—ultra-thin section. Scale bar = 1 µm (**a**,**c**,**e**,**g**) or 100 nm (**b**,**d**,**f**,**h**).

The effect of TET and COSAN.Na alone or in their combination on *S. epidermidis* CNCTC 5671 cells’ morphology was depicted after negative staining (Figure 2a,c,e,g) or ultra-thin layers preparation (Figure 2b,d,f,h) using transmission electron microscopy (TEM). 

As expected, a negative staining of control sample (Figure 2a) cultured without the presence of any of the antimicrobial agents shows undamaged coccus in grape arrangement. Very similar cells were obtained when cultured in the presence of subinhibitory concentration of 4.5 µg/mL TET (Figure 2c). There is only a hint of better staining of the cells, indicating a slightly damaged membrane. A significant difference in the cells’ morphology was found in the presence of subinhibitory concentration of COSAN.Na (2.28 µg/mL), as shown in Figure 2e. The cells were arranged in a large mass coated most probably by crystals of COSAN.Na. The combination of TET (4.5 µg/mL) and COSAN.Na (2.28 µg/mL) caused a significant inhibition of *S. epidermidis* CNCTC 5671 cells which were partially coated by crystals of COSAN.Na and partially had disrupted cells envelopes with released cell contents (Figure 2g). The ultra-thin sections of the same samples show undamaged cells in control sample (Figure 2b) as well as sample cultured in the presence of subinhibitory concentration of TET (4.5 µg/mL) (Figure 2d). On the contrary, the cells of *S. epidermidis* CNCTC 5671 affected by subinhibitory concentration of COSAN.Na (2.28 µg/mL) had disrupted cell envelopes followed by morphological changes in cells and visible degradation of intracellular space (Figure 2f). The combination of both antimicrobial agents caused complete cell lysis and leakage of intracellular contents (Figure 2h).

## 3. Discussion

The ability of COSAN.Na to inhibit planktonic growth of Gram-positive cocci was described in our previous study, Kvasničková et al. [30]. Based on a high effectivity of COSAN.Na inhibitory activity alone, we would like to combine its surfactant action with usually used antibiotics TET, ERM and VAN. For this purpose, we chose an often overlooked, but still very important, representative of opportunistic pathogenic Gram-positive bacterium *S. epidermidis*.

The MIC_80_ of ERM and VAN reached very similar values (0.5–0.75 µg/mL for ERM and 1.5–2.75 µg/mL for VAN) for all strains observed in our study, essentially the same as for COSAN.Na (2–3.1 µg/mL). This cannot be said for TET, which MIC_80_ ranged from 5 to 65 µg/mL. Based on these results, COSAN.Na can therefore be declared a more stable antimicrobial agent than TET. As for MIC determination of COSAN.Na, the concentration range of MIC_80_ confirmed in our study corresponds to the results of MIC (1–16 µg/mL) of the four derivatives of COSAN^-^ described by Romero et al. [46]. The researchers also stated that COSAN^-^ derivatives possess bactericidal rather than bacteriostatic action towards *E. faecalis* and *S. aureus*. In contrast, our data showed that COSAN.Na had bactericidal action only in the case of *S. epidermidis* DBM 3179, and it was bacteriostatic against the remaining strains. This means that bacteriostatic/bactericidal activity of COSAN-based compounds is highly genus-, species-, and strain-dependent. 

The combination of effective antibacterial agents within the amphiphilic activity with conventionally used antibiotics is a promising approach to overcoming bacterial resistance to them. Based on this consideration, we tested whether COSAN.Na enhances antibiotics TET, ERM or VAN action towards *S. epidermidis* planktonic growth. To the best of our knowledge, there is no other publication available that deals with such a topic. However, it should be noted that it has already been reported that COSAN.Na and some of the related compounds have a very high antibacterial activity against the planktonic growth of Gram-positive cocci [30,31,32,36,44]; thus, it can be used as an inhibitory agent alone. Despite this, there may be cases where a combination of antibiotics with COSAN.Na will be desirable due to their different mechanism of action. We found that, in all cases tested, the combination of antibiotics and COSAN.Na effectively inhibited the growth of *S. epidermidis* with mostly additive effect (in some cases even with a synergistic effect), and in none of the cases was an antagonistic effect monitored. COSAN.Na enhances the antibacterial effectiveness of TET, most of all tested antibiotics, and a very important finding is that the addition of COSAN.Na to TET reduced its effective inhibitory concentration against *S. epidermidis* five to ten times. In addition, we confirmed no cytotoxicity of used antibiotics and COSAN.Na (at MIC_80_ and selected subinhibitory concentrations) as well as their combination in effective inhibitory concentrations. This corresponds to the available literature, because non-cytotoxicity of COSAN^-^ or its derivatives has been also reported in other studies [34,47,48].

The mechanism of COSAN^-^ and its derivatives interaction with cells has been previously outlined in various studies using synthetic lipid membranes [22,49,50]. The studies reported that these compounds in the form of vesicles cross through cell-free artificial lipid bilayer membranes by transmembrane translocation (without causing breakdown of membrane barrier properties). Verdiá-Báguena et al. (2014) used membrane electrical capacitance measurement, and concluded that the lipid bilayer remained intact with no aqueous pores formed in the membrane. As mentioned above, the studies dealing with the interaction of COSAN-based compounds with cell tissue cultures showed that these substances are non-cytotoxic [20,48]. In addition, Tarrés et al. [20] described no immediate effect of COSAN^-^ on cell viability of a range of mammalian cell lines: HEK293, HeLa, 3T3 cells and a lymphoblastoid line THP-1. They confirmed that, in all concentrations examined, COSAN^-^ treatment has shown no signs of cell membrane disruption. On the contrary, Zheng et al. [44] found that cell membrane of MRSA is powerfully permeabilized by cobalt bis-dicarbollide alkoxy derivative K121 (at MIC 8 µg/mL) after 2 h treatment. The authors used membrane integrity test with PI (which bounds DNA of cells with damaged membrane) fluorescence activity determination. Simultaneously, they prepared scanning electron microscopy (SEM) micrographs that showed the sunken and damaged MRSA cell wall. These findings correspond to our results of PI uptake by *S. epidermidis* cells affected by COSAN.Na at a subinhibitory concentration (2.24 µg/mL), which was also confirmed by TEM images showing the *S. epidermidis* CNCTC 5671 cell envelopes disruption. The permeabilization activity of COSAN.Na also remained intact when combined with all antibiotics tested and, in addition, TEM images clearly demonstrated cell lysis leading to cell death in the case of a combination of subinhibitory concentrations COSAN.Na and TET. Thus, COSAN.Na can be considered as an effective agent for the enhancement of antibiotics TET, ERM or VAN inhibitory action, especially in the case of resistance emergence, as these antibiotics have different mechanisms of action.

## 4. Materials and Methods

### 4.1. Microorganisms

The three collection strains of *S. epidermidis* were used in this study. *S. epidermidis* DBM 3179 was kindly provided by the Department of Biochemistry and Microbiology of the University of Chemistry and Technology in Prague. *S. epidermidis* ATCC 14990 (CNCTC 5671) was acquired from the Czech National Collection of Type Cultures and *S. epidermidis* CCM 2343 was obtained from the Czech Collection of Microorganisms. The stock cultures were stored at −70 °C in 50% glycerol solution and cultured in Tryptone Soya Broth (TSB; Oxoid, Germany) aerobically overnight at 37 °C at 150 rpm before each experiment.

### 4.2. Antimicrobial Agents

COSAN.Na was prepared from 1,2-dicarba-*closo*-dodecaborane (Katchem Praha Ltd., Prague, Czech Republic) by a modified procedure described in detail in Vaňková et al. [34]. COSAN.Na was dissolved in dimethyl sulfoxide (DMSO; Carl Roth, Germany) prior to use. The amount of COSAN.Na solution was 1% of the working volume. Control samples of 1% DMSO were included in each experiment. TET, ERM and VAN were acquired from Sigma-Aldrich (Prague, Czech Republic), dissolved in TSB, and stored at 4 °C prior to use (a maximum of one week).

### 4.3. Antimicrobial Assay

The effect of antimicrobials on planktonic growth of *S. epidermidis* was determined as described in Vaňková et al. [51]. In short, the planktonic cells suspension was adjusted to OD_600nm_ = 0.1 and 30 µL was mixed with TSB medium and appropriate volume of antimicrobials (a final concentration of 0.5–100 µg/mL) to a final volume of 320 µL in each well of microtiter plate. The cultivation of cells was carried out for 24 h, at 37 °C in 100-well microtiter plates using Bioscreen C analyzer (Oy Growth Curves Ab Ltd., Helsinki, Finland). MIC_80_ of antimicrobials were determined as the lowest concentration that inhibit 80% of microbial growth. Substance-free controls were included. At the end of the cultivation, the suspension from each well was spotted onto TSB agar plates and cultured for another 24 h at 37 °C. The MBC_99.9_ of antimicrobials was determined as the lowest concentration that inhibits any visible growth of planktonic cells. The experiments were performed in triplicate. Based on MBC_99.9_/MIC_80_ ratio, the overall bactericidal or bacteriostatic action of studied compounds was evaluated (MBC_99.9_/MIC_80_ < 4.0—bactericidal, MBC_99.9_/MIC_80_ > 4.0—bacteriostatic) according to Ayala-Núñez et al. [52]. 

The interaction of COSAN.Na with antibiotics was evaluated using FICi, as described previously by Samadi et al. [53] and in detail by Maťátková et al. [54]. The procedure of the experiment was similar to the MIC_80_ determination. The COSAN.Na was studied in a final concentration range of 0.31–3.1 µg/mL, TET in a final concentration range of 6.5–65 µg/mL, ERM in a final concentration range of 0.075–0.75 µg/mL and VAN in a final concentration range of 0.15–1.5 µg/mL, corresponding to 0.1–1 × MIC_80_ values. Based on determined FICi, the effect of COSAN.Na and antibiotics combination was evaluated as synergistic (FICi 0.1–0.5), additive (FICi 0.5–1.0) or indifferent (no effective combination found in interval of FICi 0.1–1.0) according to Guo et al. [55]. For better overview, the data are depicted in three-dimensional graphs to highlight the character of the compounds interaction. Standard deviations ranged from (±) 1 to 15%.

### 4.4. Cytotoxicity Assay

The effect of antimicrobials alone and in combination on viability of HEK 293T cells was investigated as described previously by Hynek et al. [56]. In short, HEK 293T (obtained from Sigma Aldrich, Czech Republic) at a concentration of 3 × 10^5^ cells/mL were seeded in a 48-well plate in Dulbecco’s Modified Eagle Medium supplemented with 10% fetal bovine serum and cultured in 5% CO_2_ atmosphere at 37 °C. Overnight grown cells were further cultivated for 48 h in the presence of antimicrobials tested in the appropriate concentrations (4.5 and 45 µg/mL TET, 0.3 and 0.75 µg/mL ERM, 0.15 and 2 µg/mL VAN, 1.68, 2.24 and 2.8 µg/mL COSAN.Na, the combination of 4.5 µg/mL TET and 2.24 µg/mL COSAN.Na, the combination of 0.3 µg/mL ERM and 1.68 µg/mL COSAN.Na, the combination of 0.15 µg/mL VAN and 2.24 µg/mL COSAN.Na). After this treatment, resazurin (at a final concentration of 25 µg/µL) was added to each well and the cells were incubated for an additional 4 h under the conditions previously described. The fluorescence intensity of formed resorufin expressing the extent of metabolic activity of cells was measured using microplate reader (Tecan M200Pro, Männedorf, Switzerland) at 560 nm/590 nm. Experiments were performed in triplicate.

### 4.5. Propidium Iodide Uptake Assay

The effect of antimicrobials alone and in combination on cytoplasmic membrane permeabilization of *S. epidermidis* CNCTC 5671 was evaluated using PI uptake kinetic study described in detail by Kašparová et al. [8]. Briefly, the planktonic cells suspension was adjusted to OD_600nm_ = 0.7. A 50 μL aliquot was mixed with 5 µg/mL PI (Sigma-Aldrich, Czech Republic), solutions of antimicrobials in an appropriate concentrations (4.5 µg/mL TET, 0.3 µg/mL ERM, 0.15 µg/mL VAN, 1.68 and 2.24 µg/mL COSAN.Na, the combination of 4.5 µg/mL TET and 2.24 µg/mL COSAN.Na, the combination of 0.3 µg/mL ERM and 1.68 µg/mL COSAN.Na, the combination of 0.15 µg/mL VAN and 2.24 µg/mL COSAN.Na) and phosphate-buffered saline (PBS) to a final volume of 200 µL in each well of black 96-well plates (Microplate, F-BOTTOM chimney well, Greiner Bio-One International GmbH, Kremsmünster, Austria). The cells were cultured for 90 min in Infinite M200 Pro Reader (Tecan, Switzerland) at 37 °C and the fluorescence intensity was measured at 544/620 nm each 2.5 min. The experiment included both positive (cell suspension mixed with 6 µM octenidine dihydrochloride; Tokyo Chemical Industry, Tokyo, Japan) and negative control (cell suspension in PBS), and blank (PBS). The experiments were performed in triplicate. Permeabilization activity was calculated as (%) = (*I* − *I*_blank_)/(*I*_positive control_ − *I*_blank_) × 100. *I* represents the fluorescence intensity of PI in the sample containing antimicrobials, *I*_blank_ is the fluorescence intensity of PI in the blank and *I*_positive control_ is the fluorescence intensity of PI in the presence of octenidine dihydrochloride.

### 4.6. Transmission Electron Microscopy

The effect of antimicrobials alone and in combination on cell morphology of *S. epidermidis* CNCTC 5671 was visualized using TEM. The procedure used was described in detail in Kočendová et al. [57]. Briefly, the planktonic cells suspension of *S. epidermidis* affected with COSAN.Na alone or its combination with antibiotics (4.5 µg/mL TET, 2.24 µg/mL COSAN.Na, the combination of 4.5 µg/mL TET and 2.24 µg/mL COSAN.Na) was prepared as previously described in antimicrobial assay. The parallel wells of prepared samples were merged and centrifuged (multiple centrifugations at 14,000× *g* for 10 min), and finally, the obtained pellet was resuspended in 100 μL of saline. Thereafter, the cells were fixed for 24 h at 4 °C in 3% glutaraldehyde in 0.1 M cacodylate buffer (pH 7.4) and post-fixed for 1 h at 4 °C in 1% osmium tetroxide. After dehydration by an increasing gradient of ethanol, the prepared samples were embedded in Agar 100 resin. The samples were visualized as entire cells with negative staining or ultra-thin sections (70 nm) stained in saturated uranyl acetate solution in water and Reynold´s lead citrate. Parlodion carbon- coated grids were floated on the top of a 10 µL drop of sample for 5 min. The grids were washed twice on a drop of water and stained with 0.25% phosphotungstic acid (pH 7.4). The sections and grids were observed with a JEOL JEM 1011 operating at 80 kV. The figures are depicted with a scale bar 1 µm (negative staining) and 100 nm (ultra-thin sections).

## 5. Conclusions

The present study demonstrated strong antibacterial activity of amphiphilic COSAN.Na against representative of Gram-positive cocci *S. epidermidis* at concentration range comparable or lower than antibiotics TET, ERM and VAN. This promising non-cytotoxic antibacterial agent enhances inhibitory action of all antibiotics tested with additive or synergistic effect. Although the current state of knowledge of the interaction of metallacarboranes with bipolar membranes has pointed to intact translocation, we confirmed strong permeabilization activity of COSAN.Na in the case of bacterial cells and confirmed cell envelopes disruption by TEM. This mechanism of action of COSAN.Na seems appropriate for the combination with antibiotics action based on different target site. In the case of emergence of antibiotics resistance, the addition of COSAN.Na may be a suitable solution to this widespread medical problem.

## Figures and Tables

**Table 1:** Effect of antibiotics or sodium salt of cobalt bis-dicarbollide (COSAN.Na) on planktonic growth of *S. epidermidis*. TET—tetracycline; ERM—erythromycin; VAN—vancomycin; MIC_80_—minimum inhibitory concentration of antimicrobials that inhibit 80% of microbial growth; MBC_99.9_—minimum bactericidal concentration of antimicrobials that inhibit 99.9% of visible growth of planktonic cells; MBC_99.9_/MIC_80_ < 4.0—bactericidal effect, MBC_99.9_/MIC_80_ > 4.0—bacteriostatic effect; ^a^ not determined to a given concentration.

	MIC_80_ (µg/mL)				MBC_99.9_ (µg/mL)	MBC_99.9_ /MIC_80_	Inhibitory Effect
	TET	ERM	VAN	COSAN.Na	COSAN.Na	COSAN.Na	COSAN.Na
*S. epidermidis* DBM 3179	65	0.7	1.5	3.1	6.2	2	bactericidal
*S. epidermidis* CNCTC 5671	45	0.75	2	2.8	14	5	bacteriostatic
*S. epidermidis* CCM 2343	5	0.5	2.75	2	10 ^a^	more than 5	bacteriostatic

**Table 2:** Combined effect of antibiotics and sodium salt of cobalt bis-dicarbollide (COSAN.Na) on planktonic growth of *S. epidermidis*. The combination of substances with the lowest FICi and the combination of substance with the highest reduction in the required effective antibiotic concentration is included. ATB—antibiotic; TET—tetracycline; ERM—erythromycin; VAN—vancomycin; FICi—fractional inhibitory concentration index; ^a^ concentrations of both antimicrobials in combination reaching the highest FICi and combination that caused the highest reduction in effective antibiotic concentration is identical.

	c_ATB; COSAN.Na_ (µg/mL)				FICi	Combined Effect	Reduction of c_ATB_
	TET	ERM	VAN	COSAN.Na			
*S. epidermidis* DBM 3179	13			0.62	0.4	synergistic	5×
	6.5			1.24	0.5	synergistic	10×
		0.15		1.86	0.8	additive	5×
		0.075		2.48	0.9	additive	10×
			0.15	1.86	0.7	additive	10× ^a^
*S. epidermidis* CNCTC 5671	27			0.28	0.7	additive	1.5×
	4.5			2.24	0.9	additive	10×
		0.45		0.28	0.7	additive	1.5×
		0.3		1.68	1.0	additive	2.5×
			0.15	2.24	0.9	additive	10× ^a^
*S. epidermidis* CCM 2343	4			0.2	0.9	additive	1.25×
	1			1.6	1.0	additive	5×
		0.2		0.2	0.5	synergistic	2.5×
		0.1		1.6	1.0	additive	5×
			2.2	0.2	0.9	additive	1.25×
			1.65	0.8	1.0	additive	1.5×

## Data Availability

Data is contained within the article and supplementary material.

## Supplementary Materials

The following supporting information can be downloaded at: https://www.mdpi.com/article/10.3390/ph15050534/s1, Figure S1. Inhibitory activity of tetracycline (TET), erythromycin (ERM), vancomycin (VAN) and sodium salt of cobalt bis-dicarbollide (COSAN.Na) alone or in their combination against *S. epidermidis* DBM 3179 planktonic growth, Figure S2. Inhibitory activity of tetracycline (TET), erythromycin (ERM), vancomycin (VAN) and sodium salt of cobalt bis-dicarbollide (COSAN.Na) alone or in their combination against *S. epidermidis* CNCTC 5671 planktonic growth, Figure S3. Inhibitory activity of tetracycline (TET), erythromycin (ERM), vancomycin (VAN) and sodium salt of cobalt bis-dicarbollide (COSAN.Na) alone or in their combination against *S. epidermidis* CCM 2343 planktonic growth, Table S1: Cytotoxicity of tetracycline (TET), erythromycin (ERM), vancomycin (VAN), sodium salt of cobalt bis-dicarbollide (COSAN.Na) and their combination against HEK 293T cells.
