# Supplementary material for: Cobalt Bis-Dicarbollide Enhances Antibiotics Action towards Staphylococcus epidermidis Planktonic Growth Due to Cell Envelopes Disruption
Source: Pharmaceuticals (Basel). 2022 Apr 26;15(5):534. doi: 10.3390/ph15050534 (PMC9147877; doi:10.3390/ph15050534)
Supplement: Supplementary file 1 [file pharmaceuticals-15-00534-s001.zip › pharmaceuticals-1646986-supplementary.pdf]

Cobalt Bis-Dicarbollide Enhances Antibiotics Action towards *Staphylococcus epidermidis* Planktonic Growth due to Cell Envelopes Disruption

Eva Vaňková [1,*], Kristýna Lokočová [1], Petra Kašparová [1], Romana Hadravová [2], Ivana Křížová [1], Olga Maťátková [1], Jan Masák [1] and Václav Šícha [3]

[1] Department of Biotechnology, University of Chemistry and Technology Prague, Technická 5,
166 28 Prague, Czech Republic; kristynalokocova@gmail.com (K.L.);
petra.kasparova@vscht.cz (P.K.); ivana.krizova@vscht.cz (I.K.);
olga.matatkova@vscht.cz (O.M.); jan.masak@vscht.cz (J.M.)

[2] Institute of Organic Chemistry and Biochemistry of the Czech Academy of Sciences, Flemingovo náměstí 542/2, 166 10 Prague, Czech Republic;
romana.hadravova@uochb.cas.cz

[3] Chemistry Department, Faculty of Science, Jan Evangelista Purkyně University in Ústí nad Labem,
Pasteurova 15, 40096 Ústí nad Labem, Czech Republic; vaclav.sicha@ujep.cz

* Correspondence: eva.vankova@vscht.cz

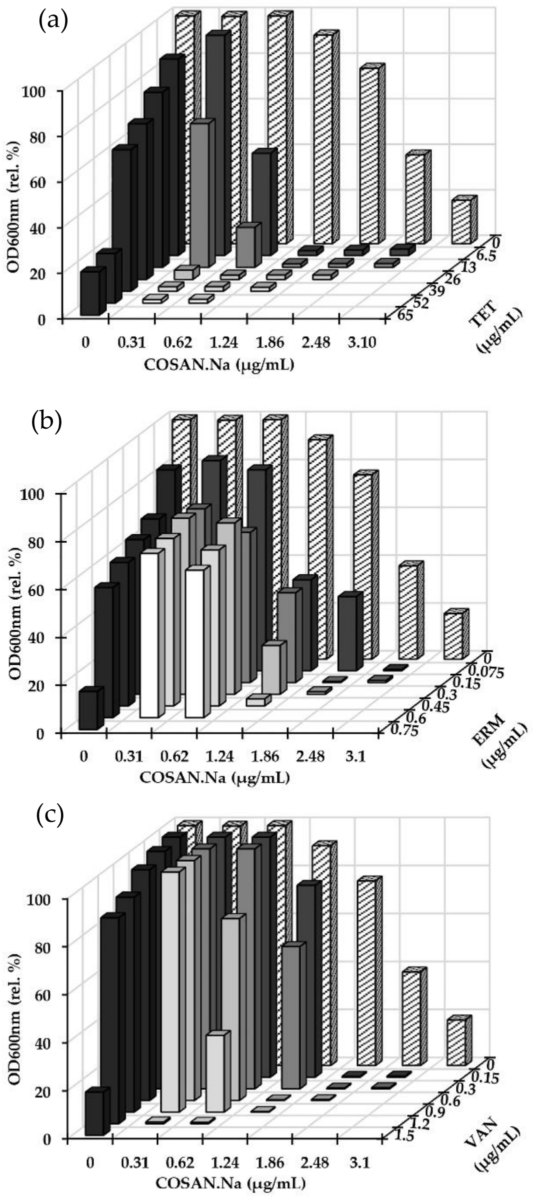

Figure S1. Inhibitory activity of tetracycline (TET), erythromycin (ERM), vancomycin (VAN) and sodium salt of cobalt bis-dicarbollide (COSAN.Na) alone or in their combination against *S. epidermidis* DBM 3179 planktonic growth. (**a**) TET + COSAN.Na; (**b**) ERM + COSAN.Na; (**c**) VAN + COSAN.Na. Results of optical density (OD) measured at 600 nm are given in relative percentages. Standard deviations ranged from (±) 1 to 15 %.

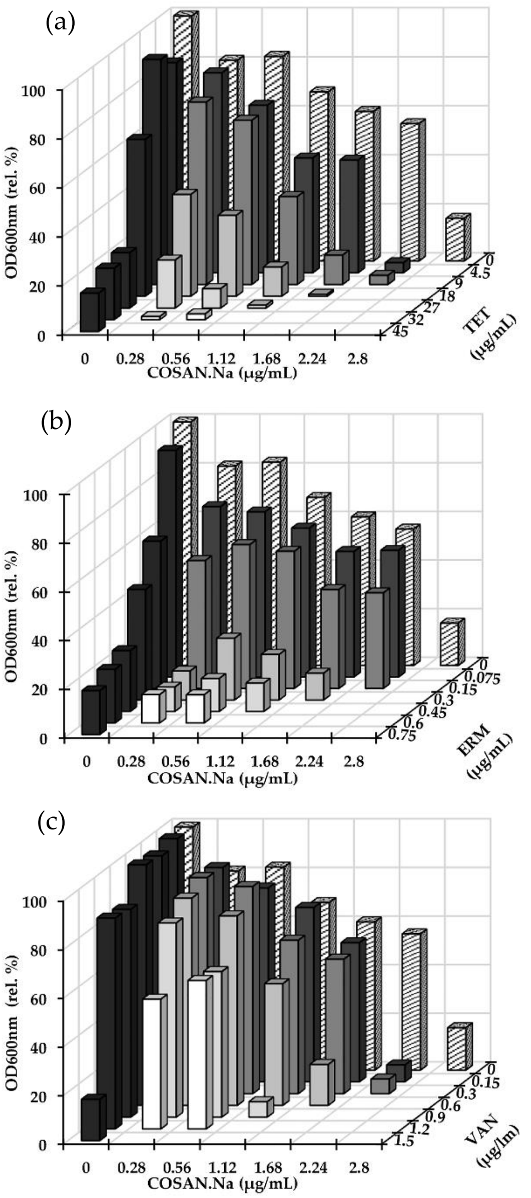

Figure S2. Inhibitory activity of tetracycline (TET), erythromycin (ERM), vancomycin (VAN) and sodium salt of cobalt bis-dicarbollide (COSAN.Na) alone or in their combination against *S. epidermidis* CNCTC 5671 planktonic growth. (**a**) TET + COSAN.Na; (**b**) ERM + COSAN.Na; (**c**) VAN + COSAN.Na. Results of optical density (OD) measured at 600 nm are given in relative percentages. Standard deviations ranged from (±) 1 to 15 %.

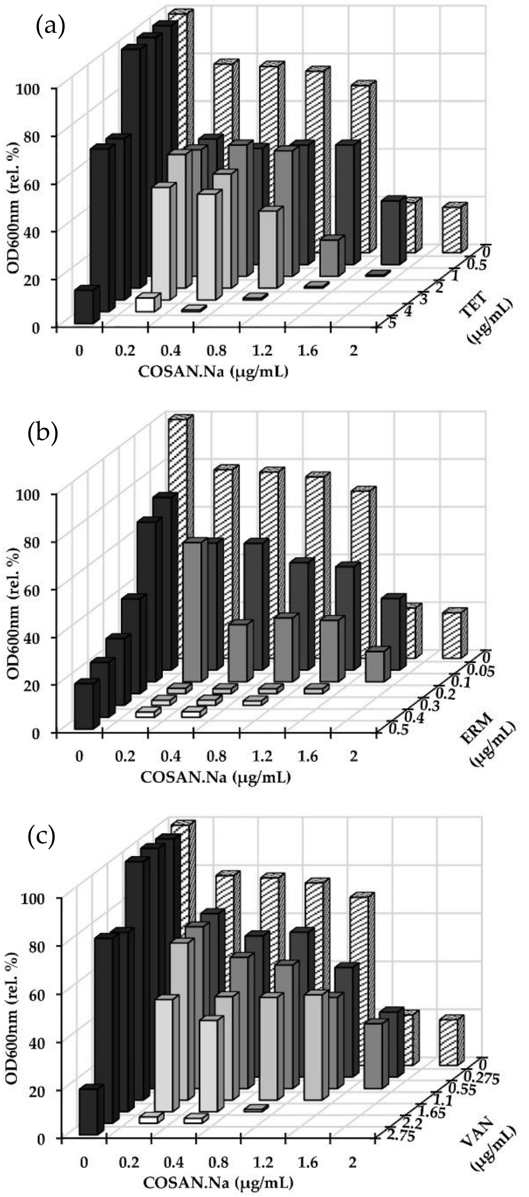

Figure S3. Inhibitory activity of tetracycline (TET), erythromycin (ERM), vancomycin (VAN) and sodium salt of cobalt bis-dicarbollide (COSAN.Na) alone or in their combination against *S. epidermidis* CCM 2343 planktonic growth. (**a**) TET + COSAN.Na; (**b**) ERM + COSAN.Na; (**c**) VAN + COSAN.Na. Results of optical density (OD) measured at 600 nm are given in relative percentages. Standard deviations ranged from (±) 1 to 15 %.

Table S1 Cytotoxicity of tetracycline (TET), erythromycin (ERM), vancomycin (VAN), sodium salt of cobalt bis-dicarbollide (COSAN.Na) and their combination against HEK 293T cells.

HEK 293T viability (rel. %)					
TET 4.5 µg/mL	TET 45 µg/mL	ERM 0.3 µg/mL	ERM 0.75 µg/mL	VAN 0.15 µg/mL	VAN 2 µg/mL
98.63	97.84	96.39	98.39	99.81	97.86
COSAN.Na 1.68 µg/mL	COSAN.Na 2.24 µg/mL	COSAN.Na 2.8 µg/mL	TET 4.5 µg/mL + COSAN.Na 2.24 µg/mL	ERM 0.3 µg/mL + COSAN.Na 1.68 µg/mL	VAN 0.15 µg/mL + COSAN.Na 2.24 µg/mL
98.77	98.00	99.09	95.58	97.85	97.26
